# Supplementary material for: The cost-of-illness trend of schizophrenia in South Korea from 2006 to 2016
Source: PLoS One. 2020 Jul 16;15(7):e0235736. doi: 10.1371/journal.pone.0235736 (PMC7365467; doi:10.1371/journal.pone.0235736)
Supplement: S1 Table — (DOCX) [file pone.0235736.s001.docx]

**S1_Table. Equations for indirect costs.**

|  | Indirect costs ($) = Q1 + Q2 + Q3 + Q4 |
| --- | --- |
| Productivity loss due to unemployment | Q1 = ∑M_jn_ × N_jn_ × (O_jn_-P_jn_); n = age, j = sex  M_jn_ = general population annual average earnings by age and sex  N_jn_ = number of persons with schizophrenia in Korea by age and sex  O_jn_ = general population employment rates by age and sex  P_jn_ = schizophrenia employment rates by age and sex |
| Productivity loss due to decreased annual earnings | Q2 = ∑M_jn_ × L_jn_ × R; n = age, j = sex  M_jn_ = general population annual average earnings by age and sex  L_jn_ = number of employed persons with schizophrenia by age and sex  R = productivity decrease ratio |
| Productivity loss due to premature death | Q3 = ∑F_jn_ × Y_jn_^t^ /(1+r)^n^; n = age, j = sex, t = age at death  F_jn_ = number of deaths attributable to schizophrenia by age and sex  Y_jn_^t^ = annual average earnings after age at death by age and sex  r = discount rate |
| Caregivers’ productivity loss | Q4 = S × C × D × E  S = number of schizophrenia patients with disability  C = percentage of those with schizophrenia that interfered with caregivers’ earnings  D = caregiving time (month)  E = general population monthly average earnings |
