# Supplementary material for: The cost-of-illness trend of schizophrenia in South Korea from 2006 to 2016
Source: PLoS One. 2020 Jul 16;15(7):e0235736. doi: 10.1371/journal.pone.0235736 (PMC7365467; doi:10.1371/journal.pone.0235736)
Supplement: Attachment — Submitted filename: Response to Reviewers.docx [file pone.0235736.s002.docx]

**Response to Reviewers**

1. At the Introduction, please include a brief description of the South Korean health care system.

- We provided a brief description of the South Korean healthcare system in the Introduction as follows:
  - *Korea provides universal healthcare through National Health Insurance (NHI) and Medical Aid program. The NHI covers about 97% of the population and the rest is covered by Medical Aid. Consequently, it is possible to construct a health insurance database that contains key data such as health records, prescriptions, etc. Moreover, the NHI Service (NHIS) provides these data to researchers for research purposes.* (pg. 4)

2. Justify the classification of costs. Usually, non-healthcare direct costs include formal and informal care; why are they considered as indirect costs?

- We included cost classifications as follows:
  - *Direct costs are disease-related payments which are divided into two parts: direct healthcare costs and direct non-healthcare costs. Indirect costs account for resources that are lost owing to the disease.* (pg. 4)
  - *Direct healthcare costs are defined as medical care costs, which include inpatient care costs, outpatient treatment costs, pharmacy costs, and non-covered care costs.* (pg. 5)
  - *Direct non-healthcare costs are defined as costs that are associated with patient management, other than direct healthcare costs.* (pg. 5)

3. Why community mental healthcare center costs are not considered health care costs?

- Community mental healthcare center costs were considered as direct non-healthcare costs owing to the Korean healthcare system:
  - *Patients do not receive a diagnosis or prescription at a community mental health center; rather, patients visit community mental healthcare centers for rehabilitation purposes. In addition, most community mental healthcare costs are covered by the government, not by the individual. Therefore, community mental healthcare center costs were considered as direct non-healthcare costs.* (pg. 6)

4. Please, provide further detail about how the results are obtained (not only about data sources, but about the procedure to arrive to final figures which are shown in the manuscript). For instance, according to reference [10], “Schizophrenia and other psychoses are associated with violence and violent offending, particularly homicide. However, most of the excess risk appears to be mediated by substance abuse comorbidity”. How do you estimate the incarceration costs due to schizophrenia? Do you assume that all the incarceration costs corresponding to schizophrenic inmates are part of non-healthcare direct costs of schizophrenia? This is only an example, more detail should be given for the rest of calculations.

- We provided a detailed formula of how each cost was calculated in the manuscript and in the Appendix:
  - *The community mental healthcare center costs, sanatoria costs, and psychiatric rehabilitation costs were estimated by the annual operating costs from each report (e.g., annual operating cost of community mental healthcare center by local mental health centers in 2007 * treated prevalence rate of schizophrenia in 2007). We estimated the transport cost by multiplying the total number of hospital visits by the mean return fare [17].* (pg. 6)
  - *For the exact equations for indirect costs, see Supporting information.* (pg. 7 and pg. 26)

5. The approach used to estimate indirect costs is the human capital approach, not the prevalence approach.

- We utilized the prevalence approach for direct costs and the human capital approach for indirect costs. We revised as follows for clarity:
  - *The cost-of-illness trend was estimated from a societal perspective using a prevalence-based approach for direct costs and a human capital approach for indirect costs.* (pg. 2)
  - *The prevalence approach was utilized for direct costs, and the human capital approach was used for indirect costs.* (pg. 4)
  - *This study was based on a societal perspective and we employed a prevalence-based approach for direct costs and a human capital approach for indirect costs.* (pg. 16)

6. Revise the Abstract, the Introduction and the Discussion sections accordingly.

- We revised the Abstract, Introduction, and the Discussion per your request.
