# Supplementary material for: The cost-of-illness trend of schizophrenia in South Korea from 2006 to 2016
Source: PLoS One. 2020 Jul 16;15(7):e0235736. doi: 10.1371/journal.pone.0235736 (PMC7365467; doi:10.1371/journal.pone.0235736)
Supplement: Attachment — Submitted filename: renamed_c6dee.docx [file pone.0235736.s003.docx]

**We greatly appreciate the academic editor and reviewer for thoroughly reviewing our manuscript. We made revisions per the comments and suggestions that were provided, which are highlighted below. We hope our responses are suitable and that the revised manuscript is now acceptable for publication in *PLoS One*.**

**Journal Requirements**

1. Please ensure that all statement are supported by adequate references, and that the references studies are critically analysed. For example, we note that your statement "Patients with schizophrenia are involved in crime more so than is the general population" does not seem to reflect the conclusions drawn by the study referenced ( Fazel, Seena, et al. "Schizophrenia and violence: systematic review and meta-analysis." PLoS medicine 6.8) , which concluded that "most of the excess risk appears to be mediated by substance abuse comorbidity". Please ensure that the aspect of substance abuse comorbidity is mentioned, to provide a balanced view on the association between schizophrenia and crime prevalence, and to reflect the content of the cited study.

- Thank you for providing this important comment. We clarified the association between schizophrenia and violence in the revised manuscript as follows:
- Patients with schizophrenia are involved in crime (mediated by substance use comorbidity) more so than is the general population [9]. **(pg. 5, lines 114–116)**
- Incarceration accounts for about 1% of the direct non-healthcare costs. However, considering the severity of the matter, efforts to prevent violence that is associated with schizophrenia are needed. A meta-analysis [9] that investigated the association between schizophrenia and violence stated that substance abuse comorbidity mediates the association between schizophrenia and violence. Therefore, experts need to consider preventing substance abuse among patients with schizophrenia. **(pg. 15, lines 278–284)**

2. In order to provide a more complete information to our readers on the topic, we would like to emphasize the importance to cross referencing very recent material on the same topic published in "PLoS ONE ". Therefore, it would be highly appreciated if you would check the contents published in the last two years of "PLoS ONE" (https://journals.plos.org/plosone/) and add all material relevant to your article to the reference list.

- We went through recent materials related to our topic in *PLoS One* and added one relevant study to our reference list, which revealed that patients with schizophrenia have a high risk of acquiring physical diseases (e.g., cardiovascular disease):
- [10] Cunningham R, Poppe K, Peterson D, Every-Palmer S, Soosay I, Jackson R. Prediction of cardiovascular disease risk among people with severe mental illness: a cohort study. PLoS One. 2019;14(9): e0221521.
